# Improvement of Surface Roughness and Hydrophobicity in PETG Parts Manufactured via Fused Deposition Modeling (FDM): An Application in 3D Printed Self–Cleaning Parts

**DOI:** 10.3390/ma12152499

**Published:** 2019-08-06

**Authors:** Juan M. Barrios, Pablo E. Romero

**Affiliations:** Department of Mechanical Engineering, University of Cordoba, Medina Azahara Avenue, 5–14071 Cordoba, Spain

**Keywords:** fused deposition modeling (FDM), polyethylene terephthalate glycol (PETG), surface roughness, sliding angle, contact angle, hydrophobicity, self-cleaning, Taguchi method, ANOVA

## Abstract

The fused deposition modeling (FDM) technique is used today by companies engaged in the fabrication of traffic signs for the manufacture of light-emitting diode LED spotlights. In this sector, the surface properties of the elements used (surface finish, hydrophobic features) are decisive because surfaces that retain little dirt and favor self–cleaning behavior are needed. A design of experiments (L27) with five factors and three levels has been carried out. The factors studied were: Layer height (LH), print temperature (T), print speed (PS), print acceleration (PA), and flow rate (F). Polyethylene terephthalate glycol (PETG) specimens of 25.0 × 25.0 × 2.4 mm have been printed and, in each of them, the surface roughness (*R_a,0_*, *R_a,90_*), sliding angle (SA_0_, SA_90_), and contact angle (CA_0_, CA_90_) in both perpendicular directions have been measured. Taguchi and ANOVA analysis shows that the most influential variables in this case are printing acceleration for *R_a, 0_* (*p*–value = 0.052) and for SA_0_ (*p*–value = 0.051) and flow rate for *R_a, 90_* (*p*–value = 0.001) and for SA_90_ (*p*–value = 0.012). Although the ANOVA results for the contact angle are not significant, specimen 8 (PA = 1500 mm/s^2^ and flow rate F = 110%) and specimen 10 (PA =1500 mm/s^2^ and F = 100%) have reached contact angle values above or near the limit value for hydrophobia, respectively.

## 1. Introduction

One pillar of industry 4.0 is additive manufacturing [1]. This technology allows [2,3]: The direct manufacture of prototypes or small batches, the production of functional parts, and the obtaining of components at a low price. There are several 3D printing techniques [4], although the most extended is the fused deposition modeling (FDM), also known as fused filament fabrication (FFF) [5].

The FDM technique consists of the orderly deposition of layers by melting a thermoplastic filament [6,7]. One of the main virtues of FDM is that it allows us to work with a wide variety of plastic materials. Polylactic acid (PLA) [8] and acrylonitrile butadiene styrene (ABS) [9] were the first materials used in FDM, but today suppliers have an extensive catalogue that includes new materials every year [10].

A recent material available on the market is polyethylene terephthalate glycol (PETG). This polyester copolymer has an increasing importance in 3D printing [11,12], due to its properties [13,14]: Durability, flexibility; high impact resistance, high chemical resistance, ultraviolet and weather resistance; low moisture absorption; it acts as a gas barrier; it is odorless, recyclable, and has food contact; it is easy to print and it does not produce fumes during the building.

**Figure 1:**
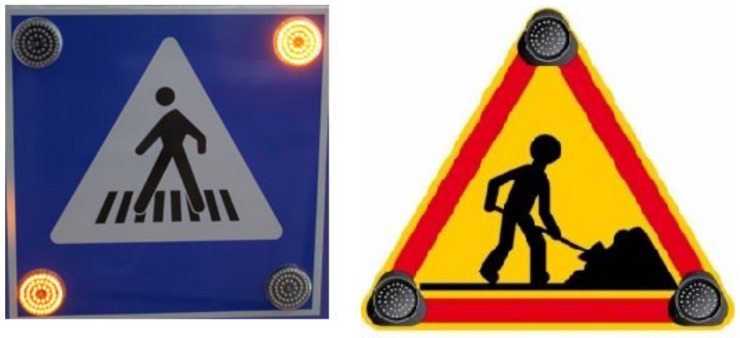
3D printed LED spotlights for road signals.

Industries dedicated to the manufacture of traffic signs use PETG to print spotlights for LEDs, due to its properties (UV and weather resistance, mainly) (Figure 1). The batch size of this type of order is usually small, and 3D printing allows manufacturing the spotlights in a cheap, immediate, and customized way. The use of 3D printing in the manufacture of elements for optics is not new [15].

In road signaling, it is important to have vertical flat elements that are not very rough (that do not accumulate dirt) and self–cleaning surfaces (when it rains, they clean themselves) [16]. In this last case, the mechanism is as follows: On hydrophobic surfaces (with low water sliding angles and high contact angles) spherical water droplets roll and carry away dust and dirt particles [17,18]. Recent works have achieved hydrophobic behavior in 3D parts printed by FDM, using a dip coating method [19].

**Figure 2:**
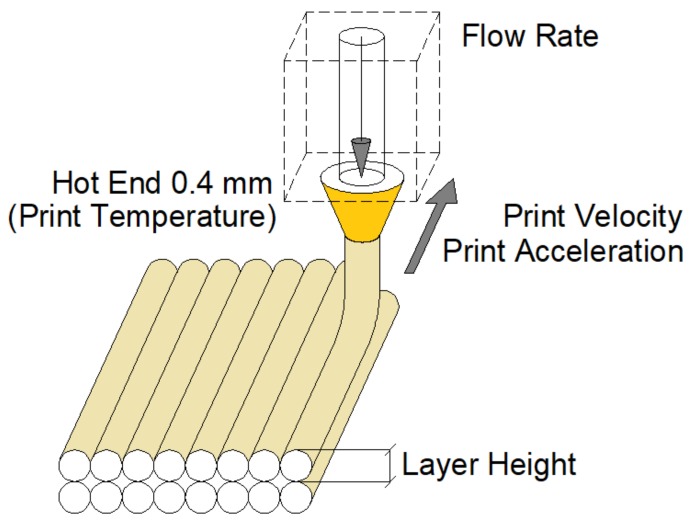
Fused deposition modeling (FDM) 3D printer parameters studied in the work.

The aim of this work is to determine the most suitable values for different printing parameters in order to reduce surface roughness and sliding angle in PETG 3D printed parts via FDM. For this purpose, an orthogonal experiment design has been carried out with five5 factors and three levels. The factors are (Figure 2): Layer height (LH), extruder temperature (T), print speed (PS), print acceleration (PA), and flow rate (F).

In FDM 3D printing, the layer height is the distance on a vertical axis between one layer and the next; the extruder temperature is defined as the temperature of the printing nozzle; the printing speed is the space traveled by the nozzle per unit of time during printing; the printing acceleration is the speed gain during printing; the flow rate is the multiplier of the filament output stream according to the part geometry [20].

## 2. Materials and Methods 

### 2.1. Design of Experiments and the CAD–CAM Process

**Figure 3:**
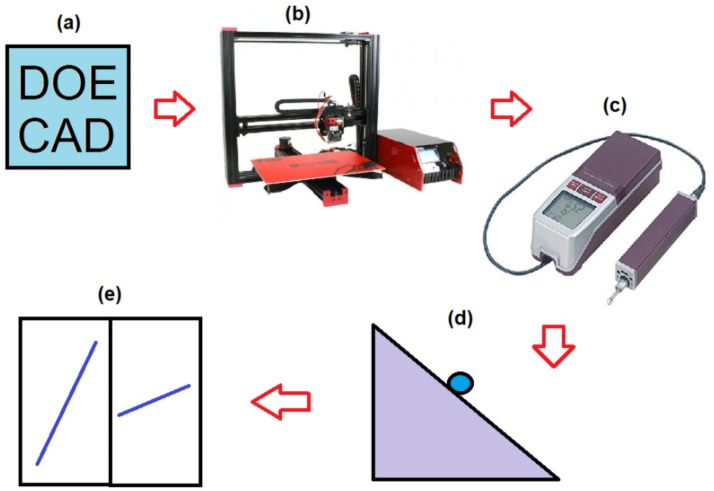
Graphical description of the methodology followed in the work: (**a**) Design of experiments and computer-aided design and computer-aided manufacturing CAD-CAM stage, (**b**) D printing of specimens, (**c**) surface roughness measurements, (**d**) sliding and contact angle measurements, (**e**) statistical data processing.

The methodology used in this work is summarized in Figure 3. An orthogonal design of experiments (DOE) was carried out [21]. The parameters studied in this DOE are shown in Table 1. One specimen of each type has been printed using the values presented in Table 2. Specimen design was performed using SolidWorks software. CURA slicing software was used to define the printing parameters planned in the DOE and to generate the numerical control (NC) code [22]. The CURA numerical control (NC) files are available online as Supplementary Materials.

### 2.2. 3D Printing

The material used in the manufacture of the specimens was PETG filament, provided by the supplier SmartMaterials 3D^®^ (SmartMaterials 3D, Alcalá la Real, Spain) [10]. The diameter of the filament used was 1.75 mm, and it was presented in spools of 750 g.

The printing system used was Tevo Black Widow Tevo Technologies^®^ (Tevo 3D printers, Zhanjiang, China) (Figure 3b). The machine had an extruder head with 0.4 mm diameter nozzle and a positioning accuracy of 0.012 mm in the *XY* plane (printing surface) and 0.004 mm in its vertical axis (*Z* axis) [23]. It was controlled by the Arduino Mega 2560 rev3 open source system [24]. A total of 27 specimens, with dimensions 25.0 × 25.0 × 2.4 mm, were printed.

The following printing parameters were set as fixed: The bed temperature was equal to room temperature; the first layer height was equal to 0.25 mm; the selected infill was grid type (rectangular); the infill percentage was equal to 50%. It should be noted that the number of shells depended on the fixed layer height: 15 layers for the layer height was equal to 0.16 mm; 12 layers for layer height was equal to 0.20 mm; 10 layers for layer height was equal to 0.24 mm.

### 2.3. Surface Roughness Measurements

**Figure 4:**
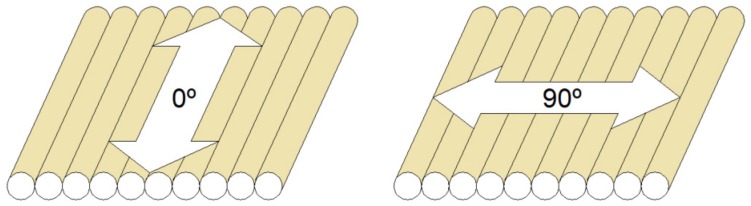
Graphical explanation of the orientations in which the measurements have been carried out.

The surface roughness (*R_a_*) [25] was measured on every printed specimen, using a perthometer MITUTOYO model SJ–210^®^ (Mitutoyo, Kawasaki, Japan) (Figure 3c) and following the standard ISO 4288: 1996 [26]. This parameter was measured 5 times along the 0° direction (*R_a,0_*) and another 5 times along the 90° direction (*R_a,90_*) in each specimen (Figure 4).

### 2.4. Sliding and Contact Angle Measurements

The sliding angle (SA) was measured by means of a low cost measurement device (Figure 3d), consisting of Arduino Uno^®^ electronics (Arduino, Torino, Italy) [24], a shield computer numerical control CNC circuit, a Nema 17 5 Kgf motor, and a DRV8825 driver. This measurement was made by depositing a drop of 50 microliters of GRIFOLS pure water (Grifols, Barcelona, Spain) on the surface of each specimen. Once deposited, the system rotates with an angular velocity of 10 microradians per second, measuring the minimum angle at which the drop moves with the help of an inclinometer HOLEX^®^ electronic protractor [27]. This inclinometer incorporates a calibration procedure that was performed before taking the measurements. The sliding angle was measured 5 times along the 0° direction (SA_0_) and another 5 times along the 90° directions (SA_90_) in each specimen (Figure 4).

The contact angle (CA) has been measured using the Attension Theta Lite device (Biolin Scientific, Gothenburg, Sweden). Drops of 2 microliters of pure GRIFOLS water were used. Each measurement was performed for a time of 100 s. Once the angle was stabilized, an instant was selected and the mean between the contact angles measured by the left and right was calculated. The contact angle was measured 5 times along the 0° direction (CA_0_) and another 5 times along the 90° directions (CA_90_) in each specimen (Figure 4).

### 2.5. Statistical Data Processing

The values of roughness and the sliding angle were analyzed using the Minitab software (Figure 3e). The analysis of the influence of each parameter on *R_a,0_*, *R_a,90_*, SA_0_, SA_90_, CA_0_, and CA_90_ was carried out according to Taguchi’s method and analysis of variance (ANOVA) [28,29,30,31].

## 3. Results

**Figure 5:**
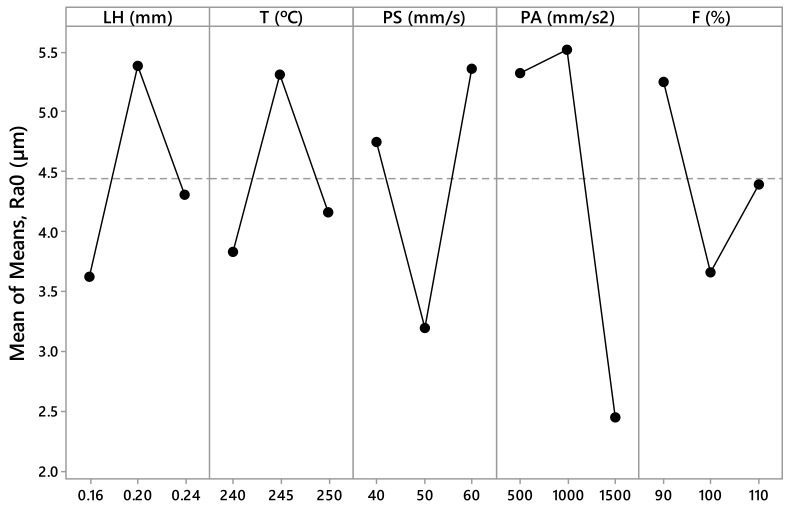
Results obtained via the Taguchi method for *R_a,0_*: Layer height (LH), printing temperature (T), printing speed (PS), printing acceleration (PA), and flow rate (F).

**Figure 6:**
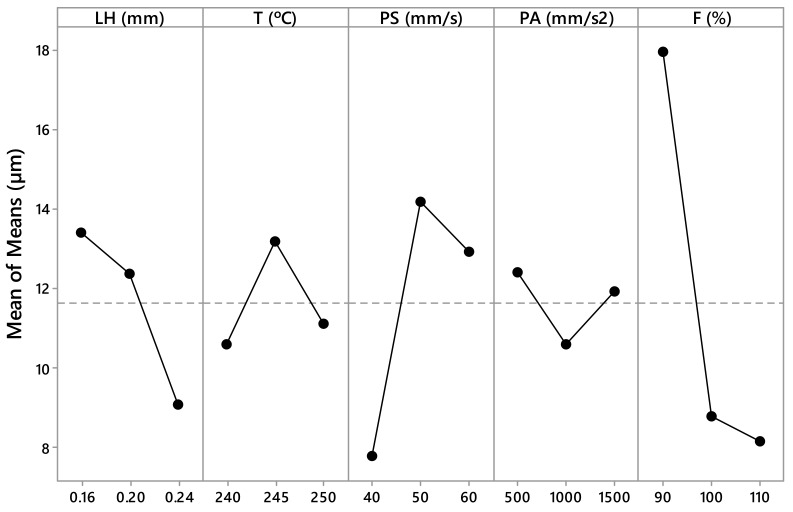
Results obtained via the Taguchi method for *R_a 90_*: Layer height (LH), printing temperature (T), printing speed (PS), printing acceleration (PA), and flow rate (F).

**Figure 7:**
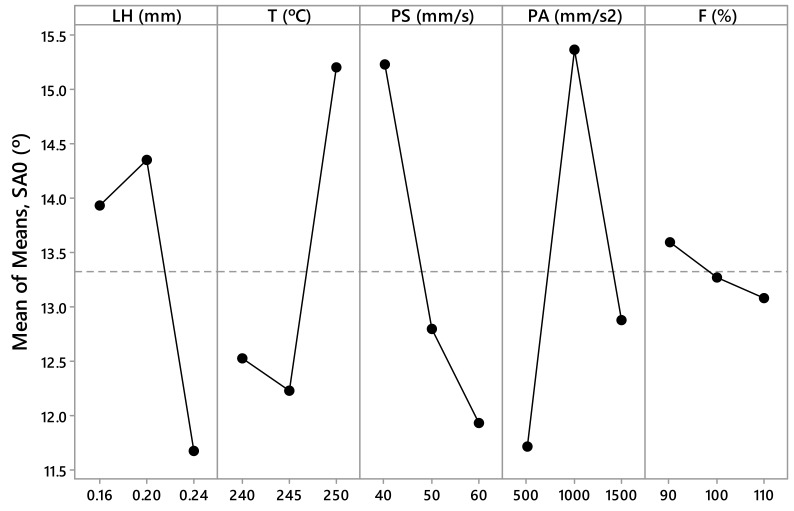
Results obtained via the Taguchi method for SA_0_ (°): Layer height (LH), printing temperature (T), printing speed (PS), printing acceleration (PA), and flow rate (F).

**Figure 8:**
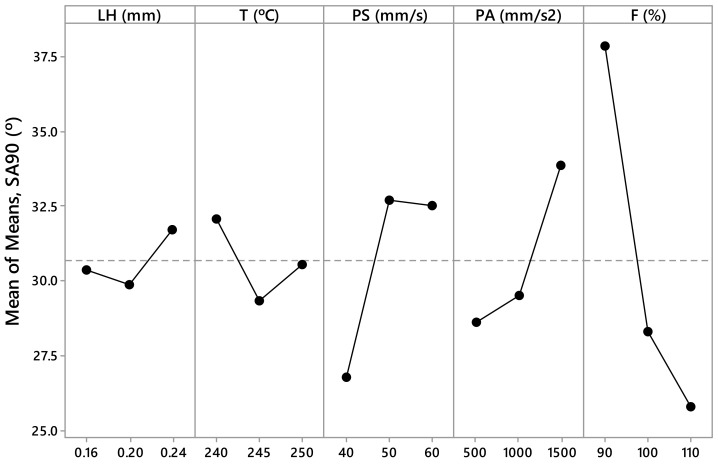
Results obtained via the Taguchi method for SA_90_ (°): Layer height (LH), printing temperature (T), printing speed (PS), printing acceleration (PA), and flow rate (F).

**Figure 9:**
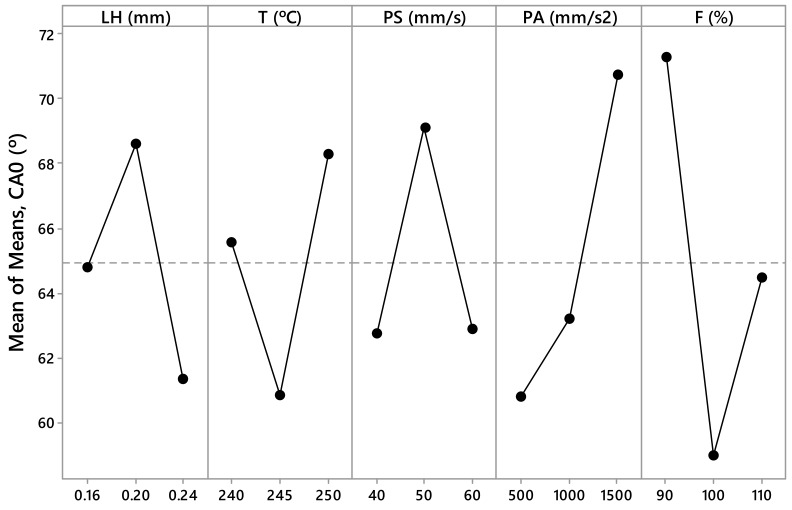
Results obtained via the Taguchi method for CA_0_ (°): Layer height (LH), printing temperature (T), printing speed (PS), printing acceleration (PA), and flow rate (F).

**Figure 10:**
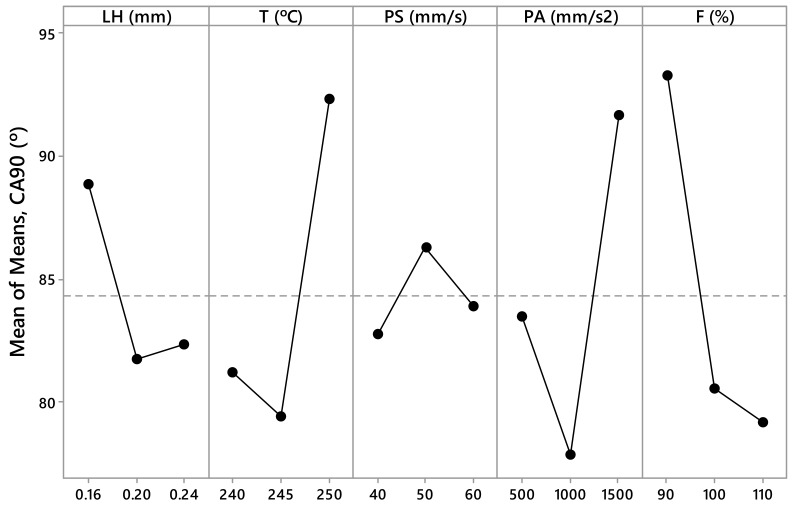
Results obtained via the Taguchi method for CA_90_ (°): Layer height (LH), printing temperature (T), printing speed (PS), printing acceleration (PA), and flow rate (F).

The measured values *R_a,0_*, *R_a,90_*, SA_0_, SA_90_, CA_0_, and CA_90_ are presented in Table 3 and Table 4. These values have been calculated as the arithmetic mean of the different measurements made for each parameter. From this data, Figure 5, Figure 6, Figure 7, Figure 8, Figure 9 and Figure 10 have been elaborated following the Taguchi method [32]. To support these results, an analysis of the variance (ANOVA) has been carried out [32].

Figure 5 shows that the most influential variables in the *R_a,0_* variation are the printing acceleration (PA). According to the ANOVA (Table 5), the parameter PA has a *p*–value equal to 0.052 (~0.05) and a contribution equal to 23%. In relation to the *R_a,90_* (Figure 6), the most influential parameters are the flow rate (F) and the printing speed (PS). According to ANOVA (Table 6), the parameter F has a *p*–value equal to 0.001 (less than 0.05) and a contribution equal to 43.74%, and the parameter PS has a *p*–value equal to 0.023 (less than 0.05) and a contribution equal to 16.81%.

In the case of the sliding angle in the direction parallel to the filament (SA_0_*)* (Figure 7), there are several influential parameters. According to the ANOVA (Table 7), the printing acceleration (PA) parameter has a *p*–value equal to 0.051 (~0.05) and a contribution of 18.36%; the printing speed (PS) parameter has a *p*–value equal to 0.076 (less than 0.10) and a contribution of 15.44%; the temperature (T) parameter has a *p*–value equal to 0.091 (less than 0.10) and a contribution of 14.23%. The most influential parameter in the variable sliding angle in the direction perpendicular to the filament (SA_90_) is the flow rate (Figure 8). According to the ANOVA (Table 8), F has a *p*–value equal to 0.012 (less than 0.05) and a contribution equal to 34.67%.

In the case of the contact angle, the PA and F parameters are the most influential factors in the case of CA_0_ (Figure 9), and the T, PA, and F parameters are the most influential in the case of CA_90_ (Figure 10). If the ANOVA is consulted, it can be seen how the F parameter has a contribution of 14.47% in CA_0_ and the PA parameter has a contribution of 10.24% in CA_0_ (Table 9). In this case, the significance is low, since the *p*–value is 0.177 and 0.282, respectively. On the other hand, the F parameter has a contribution of 15.10% in CA_90_, the PA parameter has a contribution of 12.05% in CA_90_, and the T parameter has a contribution of 12.17% in CA_90_ (Table 10). However, the significance is low, since the *p*–value is 0.148, 0.210, and 0.207, respectively.

**Figure 11:**
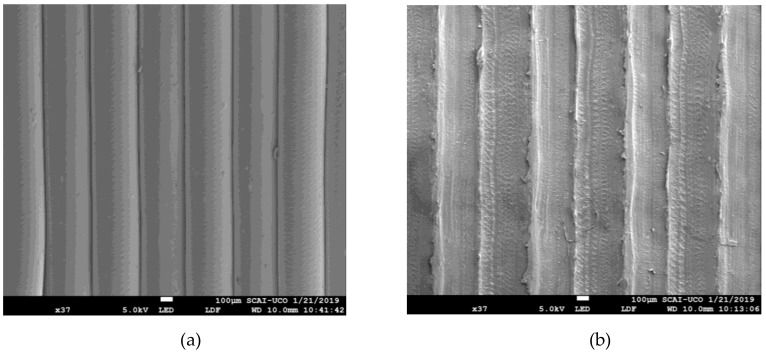
Micrographs obtained via SEM (× 37): (**a**) Specimen 22, (**b**) specimen 1.

Specimens 1 and 22 have been examined by a SEM microscope (Figure 11). As can be seen, the images support the results obtained by the experimental methodology. Specimen 1 (PA = 500 mm/s^2^; F = 90%) has high values of surface roughness (*R_a,0_* = 10.64 μm; *R_a,90_* = 12.24 μm) and sliding angle (SA_0_ = 16.76°; SA_90_ = 33.08°), and intermediate values of contact angle (CA_0_ = 68.75° and CA_90_ = 83.43°); at the other end, specimen 22 (PA = 1500 mm/s^2^; F = 100%) has low values of roughness (*R_a,0_* = 3.79 μm; *R_a,90_* = 8.68 μm) and sliding angle (SA_0_ = 14.46°; SA_90_ = 36.52°), and high values of contact angle (CA_0_ = 73.42° and CA_90_ = 105.36°). In the literature on self–cleaning surfaces [17,18], there is a preference for surfaces with low roughness (they accumulate less dirt), low sliding angles, and high contact angles (water droplets roll better).

**Figure 12:**
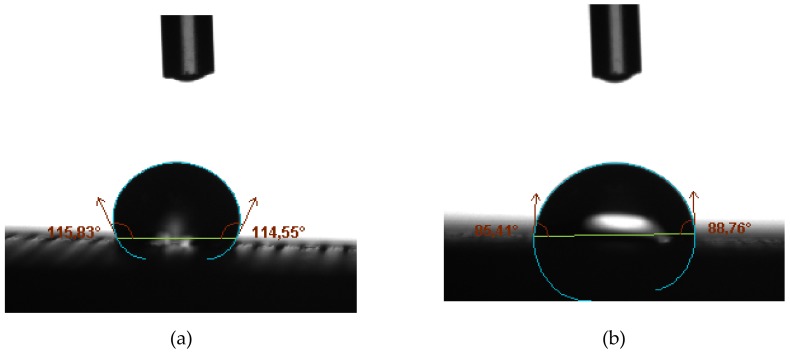
(**a**) Specimen 8: Contact angle measured in direction perpendicular to extrusion direction (CA_90_), (**b**) specimen 10: Contact angle measured in direction parallel to extrusion direction (CA_0_).

Figure 12 shows the contact angle CA_90_ for specimen 8 (PA = 1500 mm/s^2^ and F = 110%) and the contact angle CA_0_ for specimen 10 (PA = 1500 mm/s^2^ and F = 100%). Both specimens have reached the highest value for the contact angle in every direction (Table 4).

**Figure 13:**
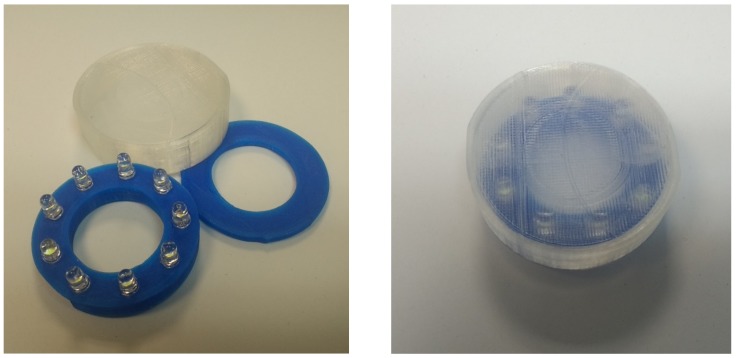
Headlight printed in polyethylene terephthalate glycol (PETG) for traffic sign.

Finally, a headlight was printed using PETG (Figure 13). This spotlight is similar to the one used by a company from Córdoba (Spain) to manufacture traffic signs with LEDs (Figure 1). In the manufacture of the diffuser, the printing parameters of test 22 have been used (it provides the most balanced values). To reduce dust accumulation (*R_a,0_ < R_a,90_*) and achieve a better self-cleaning effect (SA_0_ *<* SA_90_), the diffuser printed in 3D should be mounted with the fused filaments perpendicular to the ground.

## 4. Discussion

The influence of various printing factors on the surface properties of flat specimens made of PETG by FDM has been studied in this work. For this purpose, the surface roughness, sliding angle, and contact angle were measured in 27 specimens, manufactured according to a fractionated orthogonal arrangement. The results have been analyzed using the Taguchi method and ANOVA.

According to this, the parameters with the highest influence on surface roughness, sliding angle, and contact angle are the printing acceleration (PA) and flow rate (F) (Figure 5, Figure 6, Figure 7, Figure 8, Figure 9 and Figure 10). The contribution of PA to *R_a,0_* is 23.00% (Table 5), to SA_0_ is 18.36% (Table 7), and to CA_0_ is 10.24% (Table 9). When the PA takes the value 1500 mm/s^2^, the mean values of *R_a,0_* are lower (Figure 5), the SA_0_ values are intermediate (Figure 7), and the CA_0_ values are higher (Figure 9). On the other hand, the contribution of F to *R_a,90_* is 43.74% (Table 6), to SA_90_ is 34.67% (Table 8), and to CA_90_ is 15.10% (Table 10). When F acquires the value of 110%, the mean values of both *R_a,90_* and SA_90_ are lower (Figure 6 and Figure 8).

**Figure 14:**
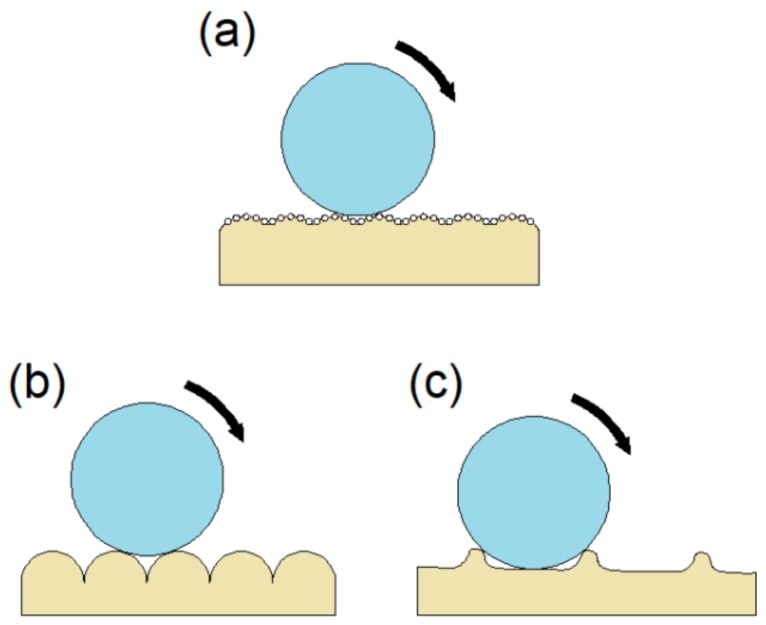
Drop sliding over different profiles: (**a**) Profile obtained by Lee et al. [19] by dip-coating in a solution with nanoparticles, (**b**) profile obtained through an appropriate selection of PA and F (specimen 22), (**c**) profile obtained through an inappropriate selection of PA and F (specimen 1).

There are not too many papers in the literature studying hydrophobicity of FDM printed parts. Lee et al. [19] have achieved sliding angles equal to 12° and contact angles equal to 150° on specimens printed in PLA using dip coating with silica nanoparticles. Nanoparticles alter the initial characteristics of the printed surface, contributing to the generation of a ‘fakir bed’. On this improved surface, the contact angle is higher (Figure 14a). However, in this work the influence of printing parameters was not studied.

In the present work, PETG FDM printed parts have been analyzed. PETG has a slightly higher contact angle than PLA [33]. In addition, the influence of printing parameters has been studied, without the need for post–processing. Based on the results obtained, low SA values are linked to the generation of rounded filament profiles (Figure 11a and Figure 14b). These profiles are obtained by modifying the road width (which is controlled by the flow rate (F) of material through the nozzle) [34,35]. Other types of profiles offer greater obstacles to the drop, which slides with difficulty (Figure 11b and Figure 14c).

**Figure 15:**
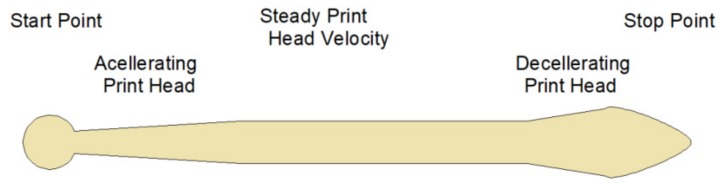
Road dimensional errors at start, acceleration, deceleration, and stopping of a print head (elaborated from [25]).

On the other hand, the influence of printing acceleration (PA) on the geometry acquired by the deposited filament has already been studied by other authors [36]. Different acceleration values mean different longitudinal geometry of the deposited material [25]. High acceleration values reduce the transition zones and allow a more homogeneous deposited filament to be obtained (Figure 15).

Finally, it should be indicated that in this work it has been possible to obtain contact angles equal to 107.91° and 86.42° for CA_90_ and CA_0_, respectively (Figure 12). These values exceed or are close to the 90° value, established as a limit to qualify a surface as hydrophobic. These values have been achieved for specimen 8 (F = 1500 mm/s^2^ and F = 110%) and specimen 10 (F = 1500 mm/s^2^ and F = 100%).

## 5. Conclusions

FDM 3D printing is increasingly used in the industry. The low cost of FDM printers and the great diversity of filament materials available on the market justify this fact. However, the printing of parts with certain surface characteristics (low surface roughness, low sliding angle, high contact angle) requires a systematic and scientific study.

In the present work, which parameters are more influential to obtain lower values of *R_a_* and SA and higher values of CA in PETG parts manufactured via FDM has been studied. The parameters studied were layer height, print temperature, print speed, print acceleration, and flow rate. A fractionated orthogonal experiment design has been used. The results were analyzed using the Taguchi method and ANOVA.

From the results obtained, it can be stated that the parameters with the greatest influence are the flow rate (F) and the print acceleration (PA). The flow rate is responsible for the fact that the section of the deposited filament is more or less circular. The print acceleration is responsible for the fact that this section is kept more or less uniform along the printing road. Depending on the programmed parameters, the profile and the behavior obtained in every specimen is different in each case.

The results obtained in this work may be of interest to companies that manufacture small batches of products that need to have certain surface characteristics. In this case, the authors have transferred the results obtained to a nearby company that manufactures traffic signs with LEDs. The diffusers of the spotlights that have been manufactured using the appropriate parameters present less surface roughness and sliding angle, and a higher contact angle. These properties make the diffuser more able to perform its function on the road.

In future works, we intend to study whether the combination of an adequate selection of printing parameters and the use of post-processing techniques (dip–coating or similar) can further improve the properties of 3D printed surfaces.

## Figures and Tables

**Table 1:** Factors and levels used in design of experiments (DOE).

Printing Parameter	Level 1	Level 2	Level 3
Layer height (LH), mm	0.16	0.20	0.24
Temperature (T), °C	240	245	250
Printing speed (PS), mm/s	40	50	60
Printing acceleration (PA), mm/s^2^	500	1000	1500
Flow rate (F), %	90	100	110

**Table 2:** Design of experiment (*L_27_*), according to the Taguchi method: Layer height (LH), printing temperature (T), printing speed (PS), printing acceleration (PA), and flow rate (F).

*No.*	*LH* *(mm)*	*T* *(* *°* *C)*	*PS* *(mm/s)*	*PA* *(mm/s^2^)*	*F* *(%)*	*No.*	*LH* *(mm)*	*T* *(* *°* *C)*	*PS* *(mm/s)*	*PA* *(mm/s^2^)*	*F* *(%)*
*1*	0.16	240	40	500	90	14	0.20	245	60	500	100
*2*	0.16	240	40	500	100	15	0.20	245	60	500	110
*3*	0.16	240	40	500	110	16	0.20	250	40	1000	110
*4*	0.16	245	50	1000	110	17	0.20	250	40	1000	90
*5*	0.16	245	50	1000	90	18	0.20	250	40	1000	100
*6*	0.16	245	50	1000	100	19	0.24	240	60	1000	110
*7*	0.16	250	60	1500	100	20	0.24	240	60	1000	90
*8*	0.16	250	60	1500	110	21	0.24	240	60	1000	100
*9*	0.16	250	60	1500	90	22	0.24	245	40	1500	100
*10*	0.20	240	50	1500	100	23	0.24	245	40	1500	110
*11*	0.20	240	50	1500	110	24	0.24	245	40	1500	90
*12*	0.20	240	50	1500	90	25	0.24	250	50	500	90
*13*	0.20	245	60	500	90	26	0.24	250	50	500	100
						27	0.24	250	50	500	110

**Table 3:** Results for surface roughness (*R_a,0_*, *R_a,90_*).

Test	*R_a,0_* (µm)		*R_a,90_* (µm)	
	*Mean*	*Std. Dev.*	*Mean*	*Std. Dev.*
1	10.64	2.22	12.24	1.24
2	0.91	0.16	6.46	0.69
3	1.12	0.21	9.16	1.55
4	2.42	0.96	32.99	0.28
5	1.80	0.77	5.50	1.12
6	8.81	1.91	10.92	1.08
7	4.55	1.96	23.65	3.27
8	1.37	0.56	14.45	1.84
9	0.95	0.17	5.41	0.32
10	1.46	0.32	23.47	1.96
11	1.66	0.54	9.05	0.78
12	1.55	0.48	10.07	0.75
13	6.25	1.54	20.08	2.65
14	7.78	1.83	15.36	4.79
15	10.17	0.75	12.56	2.00
16	9.74	0.85	10.18	1.11
17	4.69	0.95	5.46	1.39
18	5.11	0.30	5.33	1.57
19	4.27	1.07	10.66	1.99
20	6.99	2.31	8.21	0.53
21	5.86	0.59	6.05	1.28
22	3.79	0.66	8.68	0.48
23	3.05	0.49	5.72	0.57
24	3.70	1.28	6.80	0.39
25	4.12	0.73	19.65	2.16
26	4.68	1.07	8.96	0.59
27	2.25	0.14	7.12	0.47

**Table 4:** Results for sliding angle (SA_0_, SA_90_) and contact angle (CA_0_, CA_90_).

Test	SA_0_ (°)		SA_90_ (°)		CA_0_ (°)		CA_90_ (°)	
	*Mean*	*Std. Dev.*	*Mean*	*Std. Dev.*	*Mean*	*Std. Dev.*	*Mean*	*Std. Dev.*
1	16.76	2.05	33.08	2.97	68.75	5.52	83.43	11.18
2	9.40	0.93	26.31	2.75	50.84	7.64	81.03	9.32
3	14.18	1.60	18.12	3.39	57.91	4.78	85.71	3.57
4	11.24	1.69	41.93	4.52	80.33	3.49	100.42	2.81
5	15.56	2.50	18.20	2.19	30.66	0.80	52.03	7.72
6	16.28	4.13	29.91	4.55	78.65	1.40	85.95	7.98
7	12.68	0.75	39.90	2.88	77.24	3.22	107.68	6.54
8	14.64	0.83	35.88	2.09	68.24	5.60	107.91	2.10
9	14.68	3.28	30.32	4.07	70.56	2.53	96.01	6.52
10	13.00	1.29	44.32	2.63	86.42	4.28	101.50	10.38
11	11.74	2.21	27.38	3.34	66.25	1.73	71.16	6.11
12	13.08	0.94	37.94	1.41	85.33	4.43	91.29	2.97
13	15.92	2.02	46.80	3.94	70.29	1.34	98.02	6.00
14	8.33	0.92	31.40	4.41	66.70	4.91	91.29	2.96
15	16.57	1.09	26.96	0.86	38.25	11.73	37.59	7.12
16	20.29	3.87	27.10	4.82	67.39	10.23	85.15	5.38
17	20.08	3.03	24.72	5.76	72.52	3.94	87.04	1.78
18	20.24	2.55	22.38	2.49	64.44	1.92	73.09	1.81
19	10.66	0.82	34.28	1.37	42.29	3.44	62.53	4.93
20	12.36	4.27	39.60	3.69	65.92	6.09	79.86	4.67
21	11.64	3.05	27.56	2.23	66.67	13.20	74.61	2.90
22	14.46	2.10	36.52	6.41	73.42	3.53	105.36	9.58
23	12.72	3.45	19.32	1.74	53.39	7.77	64.83	4.55
24	9.02	1.21	33.25	4.65	56.03	5.47	79.60	3.20
25	7.46	1.02	36.96	3.05	75.71	8.47	95.77	3.50
26	14.72	3.84	32.08	2.14	56.46	3.99	89.78	1.87
27	12.15	2.27	25.76	3.75	62.43	4.63	88.89	3.76

**Table 5:** Results of ANOVA for *R_a,0_*.

Source	Degree of Freedom	Sequential Sums of Squares	Contribution (%)	Adjusted Mean Squares	F–Value	*p*–Value
LH (mm)	2	14.18	6.14	7.090	0.95	0.406
T (°C)	2	10.83	4.69	5.415	0.73	0.498
PS (mm/s)	2	22.36	9.68	11.182	1.50	0.252
PA (mm/s^2^)	2	53.14	23.00	26.569	3.57	0.052
F (%)	2	11.42	4.94	5.708	0.77	0.481
Error	16	119.06	51.54	7.441		
Total	26	230.99	100.00			

**Table 6:** Results of ANOVA for *R_a,90_*.

Source	Degree of Freedom	Sequential Sums of Squares	Contribution (%)	Adjusted Mean Squares	F–Value	*p*–Value
LH (mm)	2	91.94	7.44	45.972	2.12	0.152
T (°C)	2	33.42	2.70	16.709	0.77	0.479
PS (mm/s)	2	207.89	16.81	103.947	4.80	0.023
PA (mm/s^2^)	2	15.82	1.28	7.912	0.37	0.700
F (%)	2	540.90	43.74	270.451	12.48	0.001
Error	16	346.60	28.03	21.662		
Total	26	1236.58	100.00			

**Table 7:** Results of ANOVA for SA_0_.

Source	Degree of Freedom	Sequential Sums of Squares	Contribution (%)	Adjusted Mean Squares	F–Value	*p*–Value
LH (mm)	2	37.141	10.90	18.570	2.14	0.150
T (°C)	2	48.502	14.23	24.251	2.80	0.091
PS (mm/s)	2	52.624	15.44	26.312	3.04	0.076
PA (mm/s^2^)	2	62.566	18.36	31.283	3.61	0.051
F (%)	2	1.218	0.36	0.609	0.07	0.932
Error	16	138.675	40.70	8.667		
Total	26	340.727	100.00			

**Table 8:** Results of ANOVA for SA_90_.

Source	Degree of Freedom	Sequential Sums of Squares	Contribution (%)	Adjusted Mean Squares	F–Value	*p*–Value
LH (mm)	2	15.83	0.75	7.913	0.13	0.880
T (°C)	2	33.41	1.58	16.704	0.27	0.765
PS (mm/s)	2	205.87	9.76	102.934	1.68	0.218
PA (mm/s^2^)	2	142.75	6.77	71.376	1.16	0.337
F (%)	2	731.38	34.67	365.689	5.97	0.012
Error	16	980.49	46.47	61.280		
Total	26	2109.72	100.00			

**Table 9:** Results of ANOVA for CA_0_.

Source	Degree of Freedom	Sequential Sums of Squares	Contribution (%)	Adjusted Mean Squares	F–Value	*p*–Value
LH (mm)	2	237.0	5.00	118.5	0.67	0.526
T (°C)	2	257.6	5.43	128.8	0.73	0.499
PS (mm/s)	2	239.3	5.05	119.6	0.68	0.523
PA (mm/s^2^)	2	485.5	10.24	242.7	1.37	0.282
F (%)	2	685.6	14.47	342.8	1.94	0.177
Error	16	2834.3	59.81	177.1		
Total	26	4739.1	100.00			

**Table 10:** Results of ANOVA for CA_90_.

Source	Degree of Freedom	Sequential Sums of Squares	Contribution (%)	Adjusted Mean Squares	F–Value	*p*–Value
LH (mm)	2	281.43	3.88	140.71	0.55	0.585
T (°C)	2	881.62	12.17	440.81	1.74	0.207
PS (mm/s)	2	57.59	0.79	28.79	0.11	0.893
PA (mm/s^2^)	2	873.23	12.05	436.61	1.72	0.210
F (%)	2	1093.49	15.10	546.74	2.16	0.148
Error	16	4056.62	56.00	253.54		
Total	26	7243.97	100.00			

## Supplementary Materials

The CURA NC files are available online at https://www.mdpi.com/1996-1944/12/15/2499/s1.
